# Trends in local, regional and contralateral breast tumor recurrence within five years after diagnosis in the Netherlands: a population-based study including 121347 patients

**DOI:** 10.1016/j.breast.2025.104673

**Published:** 2025-12-09

**Authors:** J. Meijer, H.J.G.D. van den Bongard, L.B. Koppert, C.W. Menke-van der Houven van Oordt, L. de Munck, T.J.A. van Nijnatten, M.J.C. van der Sangen, R.J. Schipper, M.K. Schmidt, M.L. Smidt, W. Vreuls, M.C. van Maaren, S. Siesling

**Affiliations:** a Department of Research and Development, Netherlands Comprehensive Cancer Organisation (IKNL), Utrecht, the Netherlands; b Department of Health Technology and Services Research, Technical Medical Centre, University of Twente, Enschede, the Netherlands; c Department of Radiation Oncology, Amsterdam UMC, Amsterdam, the Netherlands; d Cancer Center Amsterdam, Cancer Treatment and Quality of Life / Cancer Biology and Immunology, Amsterdam, the Netherlands; e Surgery, Erasmus MC Cancer Institute, University Medical Center Rotterdam, Rotterdam, the Netherlands; f Department of Medical Oncology, Amsterdam UMC Location VUMC, Cancer Centre Amsterdam, Amsterdam, the Netherlands; g GROW Research Institute for Oncology and Reproduction, Maastricht University, Maastricht, the Netherlands; h Department of Radiology and Nuclear Medicine, Maastricht University Medical Center+, Maastricht, the Netherlands; i Department of Radiation Oncology, Catharina Hospital, Eindhoven, the Netherlands; j Department of Surgery, Catharina Hospital, Eindhoven, the Netherlands; k Division of Molecular Pathology, Netherlands Cancer Institute, Amsterdam, the Netherlands; l Department of Surgery, Maastricht University Medical Center+, Maastricht, the Netherlands; m Laboratory of Pathology East Netherlands (LabPON), Hengelo, the Netherlands

**Keywords:** Breast cancer, Local recurrence, Regional recurrence, Contralateral breast cancer, Real world data

## Abstract

**Background:**

The objective was to provide an overview of trends in five-year local recurrence (LR), regional recurrence (RR) and contralateral breast cancer (CBC) rates in the Netherlands from 2003 to 2008 and 2012–2016.

**Methods:**

Women ≥18 years diagnosed with primary early breast cancer between 2003-2008 and 2012–2016 in the Netherlands were included. LR was defined as invasive tumor on the same side as the primary breast cancer. RR was defined as regional lymph node metastasis. CBC was defined as second primary invasive breast tumor in the contralateral breast. Five-year LR, RR and CBC rates were calculated and stratified for age, stage, subtype, and grade.

**Results:**

Of the 121347 included patients, 5618 were diagnosed with a LR, RR or CBC. Five-year LR, RR and CBC rates decreased from 3.3 %, 1.9 % and 2.2 % in 2003 to 1.5 %, 1.6 % and 1.5 % in 2016, respectively, with fluctuations over the years. Slight increases were observed in RR rates for stage II-III, HR-/HER2+ and HR-/HER2-.

**Conclusion:**

(s). The observed overall decline in LR, RR and CBC rates could reflect more effective and personalized breast cancer treatment. Our results provide realistic insights in LR, RR and CBC rates and might contribute to further optimization of treatment and surveillance strategies.

## Highlights

• 5-year local recurrence rate decreased from 3.3 % in 2003 to 1.5 % in 2016.
• 5-year regional recurrence rate decreased from 1.9 % in 2003 to 1.6 % in 2016.
• 5-year contralateral breast cancer rate decreased from 2.2 % in 2003 to 1.5 % in 2016.
• 5618 patients had a LR, RR or CBC within 5 years after primary diagnosis.

## 1 Introduction

Breast cancer is the most commonly diagnosed cancer and leading cause of cancer-related deaths among women worldwide. In 2022, approximately 2.3 million new cases of breast cancer and 670000 deaths were recorded [1,2]. In the Netherlands, breast cancer is also the most commonly diagnosed cancer in women, with an increasing incidence, resulting in over 15000 new invasive breast cancer diagnoses annually [3]. Due to early detection and improved treatment [4], there has been an increase in the survival rate for women with breast cancer [3,5]. The increasing incidence and at the same time improved survival rates lead to an increasing number of patients is at risk of local recurrence (LR), regional recurrence (RR), and contralateral breast cancer (CBC). An earlier Dutch study has shown that approximately 4.7 % and 3.0 % of the breast cancer patients diagnosed in 2005 experienced a LR of RR within 10 years after primary diagnosis, respectively [6]. These percentages differ for subgroups based on age and subtype [6,7].

However, there is currently no overview of long-term trends in LR, RR and CBC rates based on more recent real world practice data. This can give insight in the burden on health care, the effect of treatment and surveillance, and has the potential to support optimization of personalized care and informed shared decision-making.

Therefore, the objective of this study is to provide a comprehensive overview of trends in LR, RR and CBC rates in patients with a history of primary invasive breast cancer without distant metastasis (DM) in the Netherlands over the periods 2003–2008 and 2012–2016.

## 2 Methods

### 2.1 Study population

Women ≥18 years diagnosed with primary invasive breast cancer without DM between 2003-2008 and 2012–2016 and surgically treated in the Netherlands were selected from the nationwide population-based Netherlands Cancer Registry (NCR). Patients with an unknown primary tumor stage or bilateral breast cancer (defined as a second tumor ≤90 days of a primary tumor) were excluded.

### 2.2 Definitions

LR was defined as an invasive tumor in the breast or chest wall on the same side as the primary breast cancer (e.g. an in-breast tumor recurrence after breast-conserving surgery, a recurrence in the chest wall or skin after mastectomy, or a second primary ipsilateral invasive breast tumor). RR was defined as a regional lymph node metastasis on the ipsilateral or contralateral side, as breast surgery may have altered the lymph node drainage [8,9]. Regional lymph nodes included either axillary, infraclavicular, supraclavicular, internal mammary or intramammary lymph node regions. CBC was defined as a second primary invasive breast tumor in the contralateral breast. The definitions are based on existing consensus-based definitions for recurrence classification [10], with the addition of regional lymph node metastasis on the contralateral side (not part of a CBC).

In this study the first event (LR, RR and/or CBC) for each patient was analysed. Patients could be classified into multiple categories if the second event occurred within 90 days, as these were considered to be synchronous.

### 2.3 Data collection (Supplementary Figure A)

Data on patient-, tumor-, and treatment-related characteristics were retrieved from the NCR, in which all newly diagnosed malignancies (including CBCs) in the Netherlands are registered. The nationwide NCR, hosted by the Netherlands Comprehensive Cancer Organisation (IKNL), is based on notification by the Dutch Nationwide Pathology Databank (Palga). Specially trained registration staff gathers data directly from patient files in all hospitals in the Netherlands. TNM was used for staging [11,12]. The Modified Bloom-Richardson Grading System was used for grading.

Data on LR and RR were additionally collected manually from the patient files by the registrars. For the 2003–2006 dataset, all patient files were searched. Data of patients diagnosed in 2007 or 2008 were available for 56 % of the Dutch hospitals. For the 2012–2016 dataset, only patient files of patients suspected of having had a LR or RR were searched. These notifications were a result of an algorithm based on codes and text in pathology reports, after linking the NCR to Palga. Completeness of LRs and RRs for the 2012–2016 dataset is estimated to be around 80 %, based on a validation study [13]. We corrected for this incompleteness by dividing the yearly number of LRs and RRs by four and then multiplying by five. The proportion of ipsilateral second primary tumors, included in the LR group, is not included in this correction, as these are complete in the NCR.

Patients with DM at time of LR, RR or CBC (diagnosed ≤90 days of the first recurrence) were included, to get an overview of all diagnosed LRs, RRs and CBCs.

### 2.4 Statistical analysis

Descriptive statistics were performed with regard to patient-, tumor-, and treatment-related characteristics. Trends over time were shown by calculating the five-year recurrence rates (LR, RR and CBC) using the dataset with correction, and stratified for age at primary diagnosis (<50, 50–69, or ≥70) [14], primary tumor stage (stage I, II, or III) and primary tumor grade (grade I, II or III). Trends for primary tumor subtype based on hormone receptor (HR) status and human epidermal growth factor receptor 2 (HER2) status (HR+/HER2-, HR+/HER2+, HR-/HER2+, or HR-/HER2-) were shown for 2005–2008 and 2012–2016 as there was no available data on subtype for patients diagnosed between 2003 and 2004. The HR + status was defined as the presence of oestrogen receptor and/or progesterone receptor positivity in ≥10 % of invasive tumor cells. Differences in average recurrence rates (for the 2003–2008 dataset compared to the 2012–2016 dataset) between datasets were tested with a two-sided unpaired *t*-test, with a *P-*value of <0.05 being considered significant.

Sensitivity analyses were performed by calculating the five-year LR, RR and CBC rates using the dataset without correction, stratified for age at primary diagnosis and primary tumor characteristics including stage, grade and subtype.

## 3 Results

### 3.1 Study population

In total, 121347 patients were included, of whom 56004 (46 %) were diagnosed between 2003 and 2008 and 65343 (54 %) between 2012 and 2016.

Over time, the incidence of primary tumor stage I increased, from 42.6 % in 2003–2008 to 51.6 % in 2012–2016. Furthermore, a significantly higher proportion of patients received systemic treatment (chemotherapy, hormonal therapy and/or targeted therapy) in 2012–2016 (68.4 %) than in 2003–2008 (58.4 %) (Table 1).

**Table 1:** Descriptive characteristics of patients diagnosed with primary invasive breast cancer without distant metastasis per dataset (N = 121347).

		2003–2008 (N = 56004)		2012–2016 (N = 65343)	
**Age** (Median, IQR) (N, %)		58	(49–69)	61	(51–69)
	<50	14462	(25.8 %)	13830	(21.2 %)
	50–69	28116	(50.2 %)	35760	(54.7 %)
	≥70	13426	(24.0 %)	15753	(24.1 %)
**Primary tumor stage** (N, %)	I	23861	(42.6 %)	33704	(51.6 %)
	II	23830	(42.6 %)	24493	(37.5 %)
	III	8313	(14.8 %)	7146	(10.9 %)
**Primary tumor grade** (N, %)	I	11527	(22.3 %)	14932	(25.1 %)
	II	23017	(44.6 %)	28309	(47.6 %)
	III	17057	(33.1 %)	16186	(27.2 %)
	Unknown	4403		5916	
**Primary tumor subtype**^a^ (N, %)	HR+/HER2-	23391	(73.7 %)	47963	(75.7 %)
	HR+/HER2+	2884	(9.1 %)	5564	(8.8 %)
	HR-/HER2+	1809	(5.7 %)	2743	(4.3 %)
	HR-/HER2-	3661	(11.5 %)	7090	(11.2 %)
	Unknown	3368		1983	
**Sentinel node biopsy**^b^ (N, %)	No	11895	(27.2 %)	10020	(15.3 %)
	Yes	31856	(72.8 %)	55323	(84.7 %)
	Unknown	12253		0	
**Axillary lymph node dissection** (N, %)	No	28168	(50.3 %)	53050	(81.2 %)
	Yes	27836	(49.7 %)	12293	(18.8 %)
**Type of local treatment** (N, %)	BCS	798	(1.4 %)	1156	(1.8 %)
	BCS + RT^c^	29451	(52.6 %)	38811	(59.4 %)
	Mastectomy^d^	18141	(32.4 %)	16453	(25.2 %)
	Mastectomy^d^ + RT^c^	7609	(13.6 %)	8882	(13.6 %)
	Incidental finding^e^	4	(0.0 %)	31	(0.0 %)
	Unknown	1		10	
**Chemotherapy** (N, %)	No	34704	(62.0 %)	38004	(58.2 %)
	Neoadjuvant only	2223	(4.0 %)	9261	(14.2 %)
	Adjuvant only	18896	(33.7 %)	17649	(27.0 %)
	Both	181	(0.3 %)	429	(0.7 %)
**Hormonal therapy** (N, %)	No	30658	(54.7 %)	28829	(44.1 %)
	Neoadjuvant only	22	(0.0 %)	72	(0.1 %)
	Adjuvant only	24873	(44.4 %)	35151	(53.8 %)
	Both	451	(0.8 %)	1291	(2.0 %)
**Targeted therapy** (N, %)	No	53116	(94.8 %)	58702	(89.8 %)
	Neoadjuvant only	15	(0.0 %)	148	(0.2 %)
	Adjuvant only	2630	(4.7 %)	4108	(6.3 %)
	Both	243	(0.4 %)	2385	(3.7 %)
**Systemic therapy** (N, %)	Chemotherapy only	663	(1.2 %)	2006	(3.1 %)
	Hormonal therapy only	17	(0.0 %)	57	(0.1 %)
	Targeted therapy only	0	(0.0 %)	2	(0.0 %)
	Combination of systemic therapies	32051	(57.2 %)	42682	(65.3 %)
	No systemic therapy	23273	(41.6 %)	20596	(31.5 %)

IQR, interquartile range; HR, hormone receptor; HER2, human epidermal growth factor receptor 2; BCS, breast-conserving surgery; RT, radiation therapy; LR, local recurrence; RR, regional recurrence; DM, distant metastases; CBC, contralateral breast cancer.

a Only data from 2005 to 2008 and 2012–2016 were included in the analyses by breast cancer subtype, as there was no available data in the NCR on breast cancer subtype for 2003–2004.

b Sentinel node dissection was not recorded for all regions in the Netherlands between 2003 and 2008.

c 104 patients were treated with neoadjuvant radiotherapy only, and thus did not receive adjuvant radiotherapy. These patients were most probably treated in study setting, like ABLATIVE and PAPBI [15,16].

d Mastectomy could be either with or without reconstruction.

e Incidental finding: if surgery is performed for a diagnosis other than cancer in the affected organ, but malignancy is detected through PA examination of the removed tissue.

### 3.2 Five-year recurrence rates

Of the 56004 included breast cancer patients in 2003–2008, at least one type of recurrence was observed in 3251 patients (5.8 %), of whom 237 patients (7.3 %) had multiple type of recurrences at the same time. Of the 65343 patients diagnosed between 2012 and 2016, 2367 (3.6 %) were diagnosed with a LR, RR or CBC, of whom 294 (12.4 %) had different types of recurrences.

In 2003–2008 and 2012–2016, 666 and 740 patients with LR and/or RR had synchronous DM, respectively. For patients with CBC, 48 patients (4.3 %) and 40 patients (4.3 %) had synchronous DM in 2003–2008 and 2012–2016, respectively (Table 2).

**Table 2:** Number and proportions of breast tumor recurrences per dataset by incidence year of primary tumor (N = 121347).

		Dataset with correction				Dataset without correction			
		2003–2008		2012–2016		2003–2008		2012–2016	
Total number of patients		56004		65343		56004		65343	
**LR/RR**^a^**(+ DM**^b^**)** (N, %)	LR	1015	(1.8 %)	540	(0.9 %)	1015	(1.8 %)	447	(0.7 %)
	RR	373	(0.7 %)	315	(0.5 %)	373	(0.7 %)	252	(0.4 %)
	LR + RR	108	(0.2 %)	180	(0.3 %)	108	(0.2 %)	144	(0.2 %)
	LR + DM	281	(0.5 %)	145	(0.2 %)	281	(0.5 %)	116	(0.2 %)
	RR + DM	290	(0.5 %)	416	(0.6 %)	290	(0.5 %)	333	(0.5 %)
	LR + RR + DM	95	(0.2 %)	179	(0.3 %)	95	(0.2 %)	143	(0.2 %)
	Total	2162	(3.9 %)	1794	(2.7 %)	2162	(3.9 %)	1435	(2.2 %)
**CBC (+ DM**^b^**)** (N, %)	CBC	1077	(1.9 %)	901	(1.4 %)	1077	(1.9 %)	901	(1.4 %)
	CBC + DM	48	(0.1 %)	40	(0.1 %)	48	(0.1 %)	40	(0.1 %)
	Total	1125	(2.0 %)	941	(1.4 %)	1125	(2.0 %)	941	(1.4 %)

LR, local recurrence; RR, regional recurrence; DM, distant metastases; CBC, contralateral breast cancer.

Patients with multiple recurrences ≤90 days of the fist recurrence were counted in multiple recurrence groups.

a The number of LRs and RRs in 2012–2016 were corrected by dividing the number of LRs and RRs in every year between 2012 and 2016 by four and then multiplying by five. The proportion of ipsilateral second primary tumors also in the LR group is not included in the correction here, as the number of second primary tumors is complete in the NCR.

b Patients who were diagnosed with synchronous DM ≤ 90 days of the first recurrence.

**Fig. 1:**
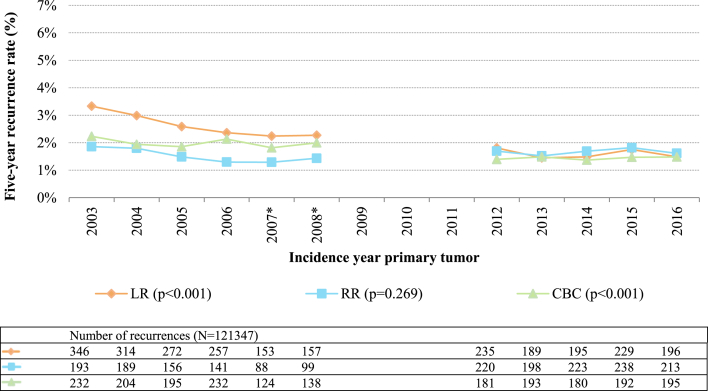
Proportions of breast tumor recurrences per incidence year of primary tumor (N = 121347). LR, local recurrence; RR, regional recurrence; CBC, contralateral breast cancer. ∗ For patients diagnosed in 2007 or 2008, data was only available from 56 % of the Dutch hospitals. Patients with multiple recurrences ≤90 days of the first recurrence were counted in multiple recurrence groups. The number of LRs and RRs in 2012–2016 were corrected by dividing the number of LRs and RRs in every year between 2012 and 2016 by four and then multiplying by five. The proportion of ipsilateral second primary tumors also in the LR group is not included in the correction here, as the number of second primary tumors is complete in the NCR. Differences in average recurrence rates (average recurrence rate for 2003–2008 dataset compared to average recurrence rate for 2012–2016 dataset) between datasets were tested with two-sided unpaired *t*-test, with a P-value of <0.05 being considered significant. Absolute percentages of recurrence per incidence year can be found in Supplementary Table 1.

Five-year LR, RR and CBC rates decreased from 3.3 %, 1.9 % and 2.2 % in 2003 to 1.5 %, 1.6 % and 1.5 % in 2016, respectively, with some fluctuations over the years (Fig. 1). Absolute percentages of recurrence per incidence year can be found in Supplementary Table A.

### 3.3 Five-year recurrence rates per age group

**Fig. 2:**
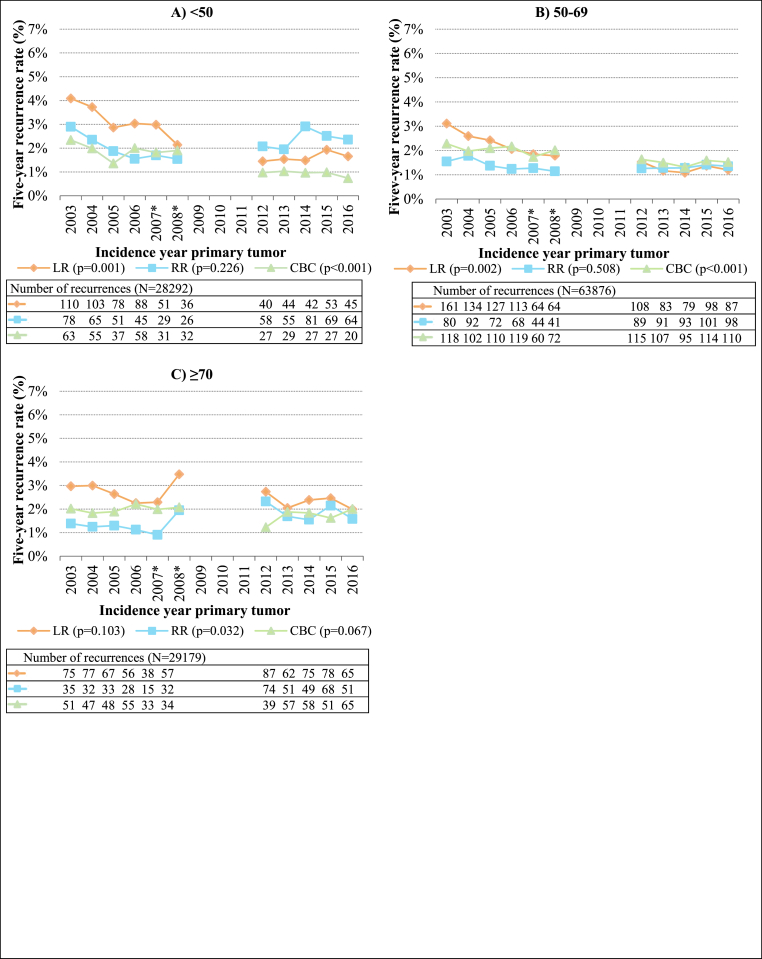
Proportions of breast tumor recurrences per incidence year of primary tumor and per age group (N = 121347). LR, local recurrence; RR, regional recurrence; CBC, contralateral breast cancer. ∗ For patients diagnosed in 2007 or 2008, data was only available from 56 % of the Dutch hospitals. Patients with multiple recurrences ≤90 days of the first recurrence were counted in multiple recurrence groups. The number of LRs and RRs in 2012–2016 were corrected by dividing the number of LRs and RRs in every year between 2012 and 2016 by four and then multiplying by five. The proportion of ipsilateral second primary tumors also in the LR group is not included in the correction here, as the number of second primary tumors is complete in the NCR. Differences in average recurrence rates (average recurrence rate for 2003–2008 dataset compared to average recurrence rate for 2012–2016 dataset) between datasets were tested with two-sided unpaired *t*-test, with a P-value of <0.05 being considered significant. Absolute percentages of recurrence per incidence year can be found in Supplementary Table 1.

For patients aged <50 (Fig. 2A), the LR and CBC rates decreased over the years, mostly during the years 2003–2008 (from 4.1 % to 2.3 % in 2003 to 1.7 % and 0.7 % in 2016, respectively). Also, for patients aged 50–69 (Fig. 2B), the LR and CBC rates decreased over the years (from 3.1 % to 2.3 % in 2003 to 1.2 % and 1.5 % in 2016, respectively). The RR rates increased over the years for patients aged ≥70 (Fig. 2C) (from 1.4 % in 2003 to 1.6 % in 2016, respectively).

Moreover, when comparing the average recurrence rates between the two datasets, the decrease in LR and CBC rates for patients aged <70 and the increase in RR rates for patients aged ≥70 showed significant differences.

### 3.4 Five-year recurrence rates per primary tumor stage

**Fig. 3:**
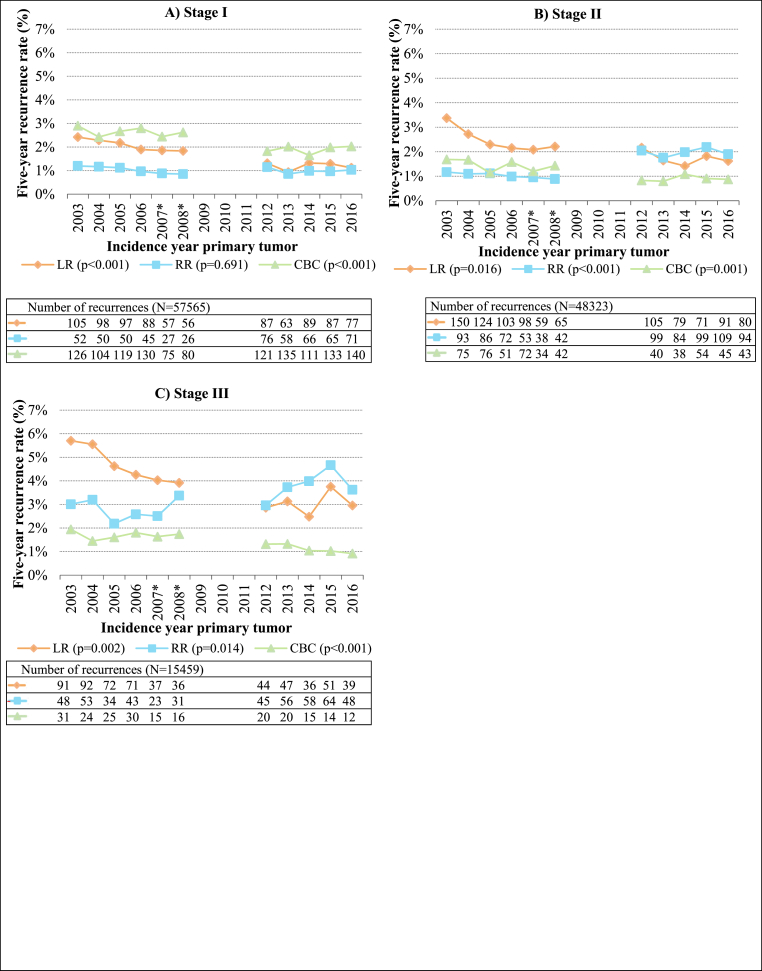
Proportions of breast tumor recurrences per incidence year of primary tumor and per primary tumor stage (N = 121347) LR, local recurrence; RR, regional recurrence; CBC, contralateral breast cancer. ∗ For patients diagnosed in 2007 or 2008, data was only available from 56 % of the Dutch hospitals. Patients with multiple recurrences ≤90 days of the first recurrence were counted in multiple recurrence groups. The number of LRs and RRs in 2012–2016 were corrected by dividing the number of LRs and RRs in every year between 2012 and 2016 by four and then multiplying by five. The proportion of ipsilateral second primary tumors also in the LR group is not included in the correction here, as the number of second primary tumors is complete in the NCR. Differences in average recurrence rates (average recurrence rate for 2003–2008 dataset compared to average recurrence rate for 2012–2016 dataset) between datasets were tested with two-sided unpaired *t*-test, with a P-value of <0.05 being considered significant. Absolute percentages of recurrence per incidence year can be found in Supplementary Table 1.

The LR rates decreased over the years for all primary tumor stages (from 2.4 %, 3.4 % and 5.7 % in 2003 to 0.9 %, 1.3 % and 2.4 % in 2016, for stage I, II and III, respectively). The RR rates remained fairly constant over the years for primary tumor stage I (Fig. 3A), while a slight increase was seen for primary tumor stage II (Fig. 3B) and III (Fig. 3C) (from 1.2 % to 3.0 % in 2003 to 1.9 % and 3.6 % in 2016, respectively). The CBC rates were slightly lower in recent years for primary stages I, II and III (from 2.9 %, 1.7 % and 1.9 % in 2003 to 2.0 %, 0.9 % and 0.9 % in 2016, respectively).

Furthermore, when comparing the average recurrence rates between the two datasets, the decrease in LR and CBC rates for all primary tumor stages was significant, while the average RR rates showed significant differences between datasets for primary tumor stages II and III.

### 3.5 Five-year recurrence rates per primary tumor grade

**Fig. 4:**
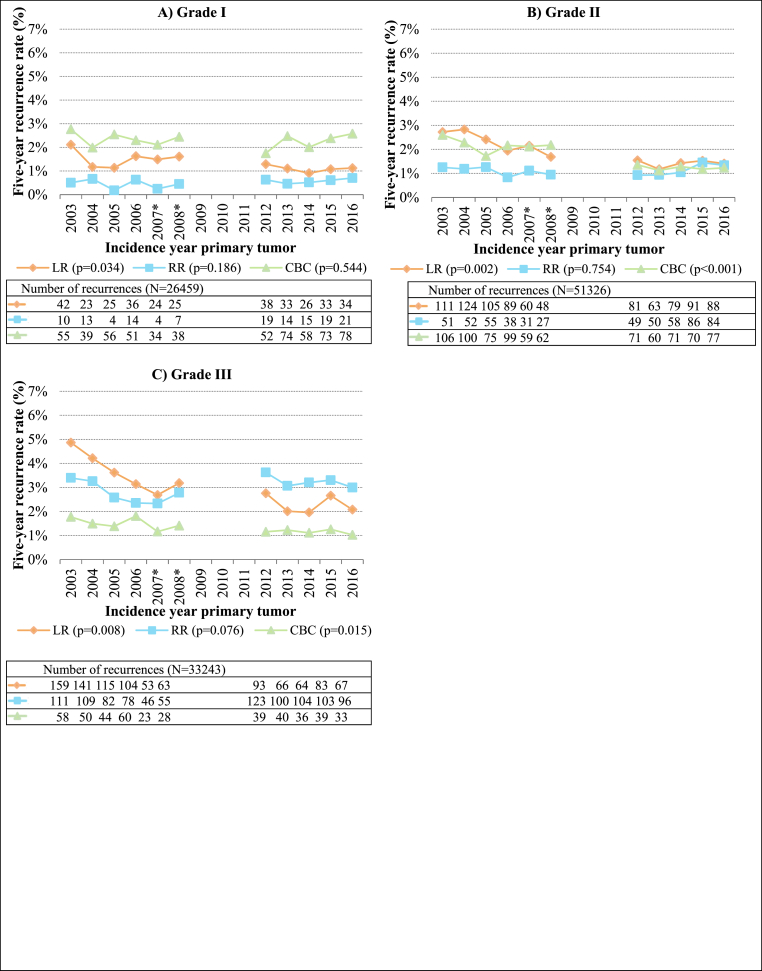
Proportions of breast tumor recurrences per incidence year of primary tumor and per primary tumor grade (N = 111,028). LR, local recurrence; RR, regional recurrence; CBC, contralateral breast cancer. ∗ For patients diagnosed in 2007 or 2008, data was only available from 56 % of the Dutch hospitals. Patients with multiple recurrences ≤90 days of the first recurrence were counted in multiple recurrence groups. The number of LRs and RRs in 2012–2016 were corrected by dividing the number of LRs and RRs in every year between 2012 and 2016 by four and then multiplying by five. The proportion of ipsilateral second primary tumors also in the LR group is not included in the correction here, as the number of second primary tumors is complete in the NCR. Differences in average recurrence rates (average recurrence rate for 2003–2008 dataset compared to average recurrence rate for 2012–2016 dataset) between datasets were tested with two-sided unpaired *t*-test, with a P-value of <0.05 being considered significant. Absolute percentages of recurrence per incidence year can be found in Supplementary Table 1.

The LR rates decreased while RR rates remained fairly constant over the years for all primary tumor grades. The CBC rates remained fairly constant over the years for primary tumor grade I (Fig. 4A), while a slight decrease was seen for primary tumor grade II (Fig. 4B) and III (Fig. 4C) (from 2.6 % to 1.8 % in 2003 to 1.2 % and 1.0 % in 2016, respectively).

Moreover, when comparing the average recurrence rates between the two datasets, the average LR and CBC rates were significantly lower for all primary tumor grades in 2012–2016 compared to 2003–2008. No significant difference in RR rates between the datasets was observed for all primary tumor grades.

### 3.6 Five-year recurrence rates per primary tumor subtype

**Fig. 5:**
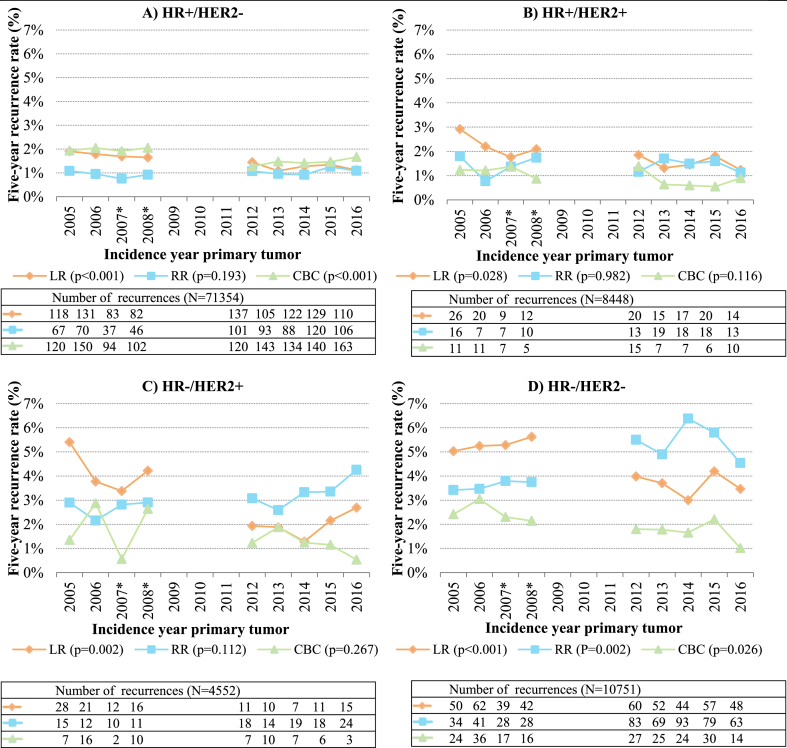
Proportions of breast tumor recurrences per incidence year of primary tumor and per primary tumor subtype (N = 95105) LR, local recurrence; RR, regional recurrence; CBC, contralateral breast cancer; HR, hormone receptor status; HER2, human epidermal growth factor receptor 2 status. ∗ For patients diagnosed in 2007 or 2008, data was only available from 56 % of the Dutch hospitals. Only data from 2005 to 2008 and 2012–2016 were included in the analyses by breast cancer subtype, as there was no available data in the NCR on breast cancer subtype for 2003–2004. Patients with multiple recurrences ≤90 days of the first recurrence were counted in multiple recurrence groups. The number of LRs and RRs in 2012–2016 were corrected by dividing the number of LRs and RRs in every year between 2012 and 2016 by four and then multiplying by five. The proportion of ipsilateral second primary tumors also in the LR group is not included in the correction here, as the number of second primary tumors is complete in the NCR. Differences in average recurrence rates (average recurrence rate for 2003–2008 dataset compared to average recurrence rate for 2012–2016 dataset) between datasets were tested with two-sided unpaired *t*-test, with a *P-*value of <0.05 being considered significant. Absolute percentages of recurrence per incidence year can be found in Supplementary Table 1.

The LR rates decreased over the years for all primary tumor subtypes (Fig. 5). The RR rates remained fairly constant over the years for all primary tumor subtypes, except for HR-/HER2-tumors where an increase was seen over the years. The RR rate for HR-/HER2-tumors increased from 3.4 % in 2005 to 6.4 % in 2014, and decreased to 4.5 % in 2016.

The CBC rates remained fairly constant over the years for primary tumor subtypes HR+/HER2+ and HR-/HER2+, while a slight decrease was seen for primary tumor subtypes HR+/HER2-and HR-/HER2- (from 1.9 % to 2.4 % in 2005 to 1.7 % and 1.0 % in 2016, respectively).

Furthermore, when comparing the average recurrence rates between the two datasets, the decrease in LR rates for all primary tumor subtypes showed significant differences, while the average RR rates showed significant differences between datasets for primary tumor subtype HR-/HER2-, and CBC rates showed significant differences between datasets for primary tumor subtypes HR+/HER2-and HR-/HER2-.

Additionally, recurrence rates were lower for patients with a primary HR + tumor in comparison to recurrence rates for patients with a primary HR-tumor.

Results of the analyses on the original data without correction are presented in Table B and Supplementary Figures B–F. The data without correction exhibited comparable trends to those observed in the dataset with correction.

## 4 Discussion

This study provides a comprehensive overview of five-year LR, RR and CBC rates in women diagnosed with primary invasive breast cancer without DM and surgically treated between 2003-2008 and 2012–2016 in the Netherlands, using population-based data of 121347 women from the NCR. LR, RR and CBC rates decline over time, from 3.3 %, 1.9 % and 2.2 % in 2003 to 1.5 %, 1.6 % and 1.5 % in 2016, respectively.

This can have multiple causes. First, a combination of multiple types of (neo)adjuvant systemic therapies was more often administered to patients diagnosed between 2012 and 2016 compared to patients diagnosed between 2003 and 2008. Second, increased personalization of systemic therapies could also contribute to the decline. For example the introduction of adjuvant trastuzumab in 2005 after trials showed improved tumor control among HER2+ breast cancer [17,18]. Lastly, another explanation could be improvements in diagnostic imaging, which enables more precise patient selection for breast-conserving therapy (BCT). For instance, the transition from analogue to digital screening mammography between 2008 and 2012 [19], and the increased utilization of MRI for screening purposes [20,21] leading to a lower proportion of higher stages observed in this study.

We revealed the most pronounced decline in LR rates among women under the age of 50, particularly between 2003 and 2008. Young women generally present with more aggressive, advanced stage tumors, which are more often HER2+ or triple negative, leading to a worse prognosis [22,23]. Consequently, it is reasonable that any improvement in treatment will yield the most significant gains in LR rates in this age group.

Moreover, results of this study showed that recurrence rates were lower for patients with a primary HR + tumor in comparison to recurrence rates for patients with a primary HR-tumor according to previous results [6].

Furthermore, a slight increase in RR rates was observed, predominantly among patients diagnosed with primary tumor stage II or III, or triple negative breast cancer. A possible explanation for these findings could be that axillary treatment de-escalation strategies, proven to be effective in low-risk breast cancer patients [[24], [25], [26], [27]], are applied to patients with higher-risk breast cancer as well. Randomized controlled trials demonstrated that a sentinel lymph node biopsy (SLNB) or axillary radiotherapy is safe and can be used as an alternative to axillary lymph node dissection (ALND) [[24], [25], [26], [27]]. Comparable axillary control and less morbidity for patients diagnosed with clinical T1-2, N0, M0 breast cancer who were treated with BCT and SLNB alone without ALND were found. Ongoing trials, such as the NRG Oncology/NSABP B-51/RTOG 1304 trial and the Alliance A011202 trial, aim to gain a deeper understanding of whether further de-escalation of axillary treatment is also safe for patients diagnosed with clinical T1-3, N1, M0 breast cancer [28,29]. However, it is possible that the results of these trials may also have been applied to patients with higher-risk breast cancer as well, without any evidence that this compromises oncological outcomes.

In general, LR, RR and CBC rates were low, which is reassuring in the current era of more effective, personalized and de-escalated breast cancer care in the Netherlands. For instance, there has been a gradual transition in surgical procedures in the Netherlands from mastectomy to BCT [[30], [31], [32]]. Our data indeed showed that more BCT was administered in 2012–2016, as compared to 2003–2008. Moreover, the increased administration of NST may have contributed to down-staging of the tumor and therewith the increase in BCT [[33], [34], [35], [36]]. Furthermore, around 2010, there has been a transition to moderate hypofractionated radiotherapy (shorter schedules with lower total dose) [37,38], and for patients with low-risk tumors from whole-breast to partial-breast radiotherapy [[39], [40], [41]] in the Netherlands.

### 4.1 Strengths and limitations

To the best of our knowledge, this is the first study that included a large number of patients with five-year follow-up data over such a long time period. A strength of this study is its population-based design, which provides a representative reflection of patients seen in daily practice.

A limitation of this study is that no data were available for the years 2009–2011. However, due to the large time period covered in this study, we were still able to analyse time trends. Another important limitation of this study is that the incidence of LR and RR was estimated to be incomplete for approximately 20 % in the 2012–2016 dataset of which half of the missed LRs and RRs could be attributed to only clinical detection (Palga only contains pathologically confirmed diagnoses) and the other half to incomplete or incorrect information in pathology reports. This missingness was assumed to be random across subgroups [42]. Since we corrected for the missing data, overall conclusions about trends over time are assumed to be valid. Sensitivity analyses on the original data without correction revealed the same trends. This may be attributed to the fact that the absolute number of events is already low, thereby limiting the change in percentages. Lastly, data of patients diagnosed in 2007 or 2008 were available for 56 % of the Dutch hospitals due to budget restraints, but this was a representative reflection of the total population so should not have affected the observed time trends.

### 4.2 Clinical implications

Despite the current era of de-escalation in breast cancer treatment over the years, the Netherlands continues to experience low levels of LR, RR and CBC rates. The rates varied over subgroups and combining tumour characteristics for risk estimation, as can be performed in the INFLUENCE nomogram, can be used to support decision-making concerning the optimal surveillance schedule [43]. Risk estimations can reduce fear of recurrence and improve shared decision-making [44]. In cases where follow-up is deemed less necessary due to a low risk profile for recurrences, the frequence of monitoring may even be further reduced as currently is investigated in the ongoing NABOR study, which focusses on the (cost-)effectiveness of personalized follow-up [45].

Furthermore, insight in the trends and simply identifying patients with LR, RR or CBC can support future research on treatment and follow-up of this patient group. This could improve clinical decision-making, expertise and collaboration between hospitals and improve the quality of care in breast cancer patients. Moreover, it can support capacity planning.

## 5 Conclusion

Despite the de-escalation in breast cancer treatment over the years, the Netherlands continues to experience low levels and a slight decrease of LR, RR and CBC rates. This reflects effective, personalized and high quality of primary breast cancer care in the Netherlands. LR, RR and CBC rates declined or remained stable in different prognostic subgroups over time in women with primary invasive breast cancer diagnosis without DM over 2003–2008 and 2012–2016. This may be a reflection of improvements in diagnostic imaging and more effective, personalized breast cancer treatment procedures in the Netherlands. A slight increase in RR rates was observed in patients with primary tumor stage II, stage III, or primary tumor subtype HR-/HER2-. However, the absolute RR rate remained low even for these groups. These results provide both patients and clinicians with realistic insights in LR, RR and CBC rates, which might contribute to further optimization of treatment and surveillance strategies of breast cancer patients.

## CRediT authorship contribution statement

**J. Meijer:** Conceptualization, Formal analysis, Investigation, Visualization, Writing – original draft. **H.J.G.D. van den Bongard:** Conceptualization, Supervision, Writing – review & editing. **L.B. Koppert:** Conceptualization, Supervision, Writing – review & editing. **C.W. Menke-van der Houven van Oordt:** Writing – review & editing. **L. de Munck:** Writing – review & editing. **T.J.A. van Nijnatten:** Writing – review & editing. **M.J.C. van der Sangen:** Writing – review & editing. **R.J. Schipper:** Writing – review & editing. **M.K. Schmidt:** Writing – review & editing. **M.L. Smidt:** Writing – review & editing. **W. Vreuls:** Writing – review & editing. **M.C. van Maaren:** Conceptualization, Data curation, Investigation, Supervision, Writing – review & editing. **S. Siesling:** Conceptualization, Supervision, Writing – review & editing.

## Declaration of competing interests

Given their role as special editor, Sabine Siesling had no involvement in the peer-review of this article and has no access to information regarding its peer-review. Full responsibility for the editorial process for this article was delegated to another journal editor.

## Data Availability

The dataset(s) supporting the conclusions of this article is(are) included within the article (and its additional file(s)).
