# Supplementary material for: Trends in local, regional and contralateral breast tumor recurrence within five years after diagnosis in the Netherlands: a population-based study including 121347 patients
Source: Breast. 2025 Dec 9;85:104673. doi: 10.1016/j.breast.2025.104673 (PMC12765441; doi:10.1016/j.breast.2025.104673)
Supplement: Multimedia component 1 [file mmc1.docx]

**Supplementary Materials**

**Supplementary Table A:** Absolute percentages of recurrences per incidence year of primary tumor and per age group, primary tumor stage, grade and subtype (N=121347).

|  |  | **2003-2008** (N=56004) | | | | | | **2012-2016** (N=65343) | | | | |
| --- | --- | --- | --- | --- | --- | --- | --- | --- | --- | --- | --- | --- |
|  |  | **2003** | **2004** | **2005** | **2006** | **2007*** | **2008*** | **2012** | **2013** | **2014** | **2015** | **2016** |
| **Total** | LR RR CBC | 3.3%  1.9%  2.2% | 3.0%  1.8%  1.9% | 2.6%  1.5%  1.9% | 2.4%  1.3%  2.1% | 2.2%  1.3%  1.8% | 2.3%  1.4%  2.0% | 1.8%  1.7%  1.4% | 1.5%  1.5%  1.5% | 1.5%  1.7%  1.4% | 1.8%  1.8%  1.5% | 1.5%  1.6%  1.5% |
| **Age groups**  **<50** | LR RR CBC | 4.1%  2.9%  2.3% | 3.7%  2.4%  2.0% | 2.9%  1.9%  1.4% | 3.0%  1.6%  2.0% | 3.0%  1.7%  1.8% | 2.1%  1.6%  1.9% | 1.5%  2.1%  1.0% | 1.5%  2.0%  1.0% | 1.5%  2.9%  1.0% | 1.9%  2.5%  1.0% | 1.7%  2.4%  0.7% |
| **50-69** | LR RR CBC | 3.1%  1.6%  2.3% | 2.6%  1.8%  2.0% | 2.4%  1.4%  2.1% | 2.1%  1.2%  2.2% | 1.9%  1.3%  1.7% | 1.8%  1.1%  2.0% | 1.5%  1.3%  1.6% | 1.2%  1.3%  1.5% | 1.1%  1.3%  1.3% | 1.4%  1.4%  1.6% | 1.2%  1.4%  1.5% |
| **≥70** | LR RR CBC | 3.0%  1.4%  2.0% | 3.0%  1.2%  1.8% | 2.6%  1.3%  1.9% | 2.3%  1.1%  2.2% | 2.3%  0.9%  2.0% | 3.5%  2.0%  2.1% | 2.7%  2.3%  1.2% | 2.0%  1.7%  1.9% | 2.4%  1.5%  1.8% | 2.5%  2.1%  1.6% | 2.0%  1.6%  2.0% |
| **Stage**  **I** | LR RR CBC | 2.4%  1.2%  2.9% | 2.3%  1.2%  2.4% | 2.2%  1.1%  2.7% | 1.9%  1.0%  2.8% | 1.9%  0.9%  2.4% | 1.8%  0.9%  2.6% | 1.3%  1.1%  1.8% | 0.9%  0.9%  2.0% | 1.3%  1.0%  1.6% | 1.3%  1.0%  2.0% | 1.1%  1.0%  2.0% |
| **II** | LR RR CBC | 3.4%  1.2%  1.7% | 2.7%  1.1%  1.7% | 2.3%  1.1%  1.1% | 2.2%  1.0%  1.6% | 2.1%  1.0%  1.2% | 2.2%  0.9%  1.4% | 2.2%  2.0%  0.8% | 1.6%  1.8%  0.8% | 1.4%  2.0%  1.1% | 1.8%  2.2%  0.9% | 1.6%  1.9%  0.9% |
| **III** | LR RR CBC | 5.7%  3.0%  1.9% | 5.6%  3.2%  1.4% | 4.6%  2.2%  1.6% | 4.3%  2.6%  1.8% | 4.0%  2.5%  1.6% | 3.9%  3.4%  1.7% | 2.9%  3.0%  1.3% | 3.1%  3.7%  1.3% | 2.5%  4.0%  1.0% | 3.8%  4.7%  1.0% | 3.0%  3.6%  0.9% |
| **Grade**  **I** | LR RR CBC | 2.1%  0.5%  2.8% | 1.2%  0.7%  2.0% | 1.1%  0.2%  2.5% | 1.6%  0.6%  2.3% | 1.5%  2.5%  2.1% | 1.6%  0.5%  2.4% | 1.3%  0.6%  1.8% | 1.1%  0.5%  2.5% | 0.9%  0.5%  2.0% | 1.1%  0.6%  2.4% | 1.1%  0.7%  2.6% |
| **II** | LR RR CBC | 2.7%  1.3%  2.6% | 2.8%  1.2%  2.3% | 2.4%  1.3%  1.7% | 1.9%  0.8%  2.2% | 2.2%  1.1%  2.1% | 1.7%  0.9%  2.2% | 1.5%  0.9%  1.4% | 1.2%  0.9%  1.1% | 1.4%  1.0%  1.3% | 1.5%  1.5%  1.2% | 1.4%  1.3%  1.2% |
| **III** | LR RR CBC | 4.9%  3.4%  1.8% | 4.2%  3.3%  1.5% | 3.6%  2.6%  1.4% | 3.1%  2.4%  1.8% | 2.7%  2.3%  1.2% | 3.2%  2.8%  1.4% | 2.8%  3.6%  1.2% | 2.0%  3.1%  1.2% | 2.0%  3.2%  1.1% | 2.7%  3.3%  1.3% | 2.1%  3.0%  1.0% |
| **Subtype**  **HR+/HER2-** | LR RR CBC | -  -  - | -  -  - | 1.9%  1.1%  1.9% | 1.8%  1.0%  2.0% | 1.7%  1.0%  2.0% | 1.7%  0.9%  2.1% | 1.4%  1.1%  1.3% | 1.1%  1.0%  1.5% | 1.3%  0.9%  1.4% | 1.3%  1.3%  1.5% | 1.1%  1.1%  1.7% |
| **HR+/HER2+** | LR RR CBC | -  -  - | -  -  - | 2.9%  1.8%  1.2% | 2.2%  0.8%  1.2% | 1.8%  1.4%  1.4% | 2.1%  1.7%  0.9% | 1.9%  1.2%  1.4% | 1.3%  1.7%  0.6% | 1.5%  1.5%  0.6% | 1.8%  1.6%  0.5% | 1.2%  1.1%  0.9% |
| **HR-/HER2+** | LR RR CBC | -  -  - | -  -  - | 5.4%  2.9%  1.4% | 3.8%  2.2%  2.9% | 3.4%  2.8%  0.6% | 4.2%  2.9%  2.6% | 1.9%  3.1%  1.2% | 1.9%  2.6%  1.9% | 1.3%  3.3%  1.2% | 2.2%  3.4%  1.1% | 2.7%  4.3%  0.5% |
| **HR-/HER2-** | LR RR CBC | -  -  - | -  -  - | 5.0%  3.4%  2.4% | 5.2%  3.5%  3.0% | 5.3%  3.8%  2.3% | 5.6%  3.7%  2.1% | 4.0%  5.5%  1.8% | 3.7%  4.9%  1.8% | 3.0%  6.4%  1.7% | 4.2%  5.8%  2.2% | 3.5%  4.5%  1.0% |
| LR, local recurrence; RR, regional recurrence; CBC, contralateral breast cancer; HR, hormone receptor status; HER2, human epidermal growth factor receptor 2 status.  ***** For patients diagnosed in 2007 or 2008, data was only available from 56% of the Dutch hospitals.  Only data from 2005-2008 and 2012-2016 were included in the analyses by breast cancer subtype, as there was no available data in the NCR on breast cancer subtype for 2003-2004.  Patients with multiple recurrences ≤90 days of the first recurrence were counted in multiple recurrence groups.  The number of LRs and RRs in 2012-2016 were corrected by dividing the number of LRs and RRs in every year between 2012 and 2016 by four and then multiplying by five. The proportion of ipsilateral second primary tumors also in the LR group is not included in the correction here, as the number of second primary tumors is complete in the NCR. | | | | | | | | | | | | |

| 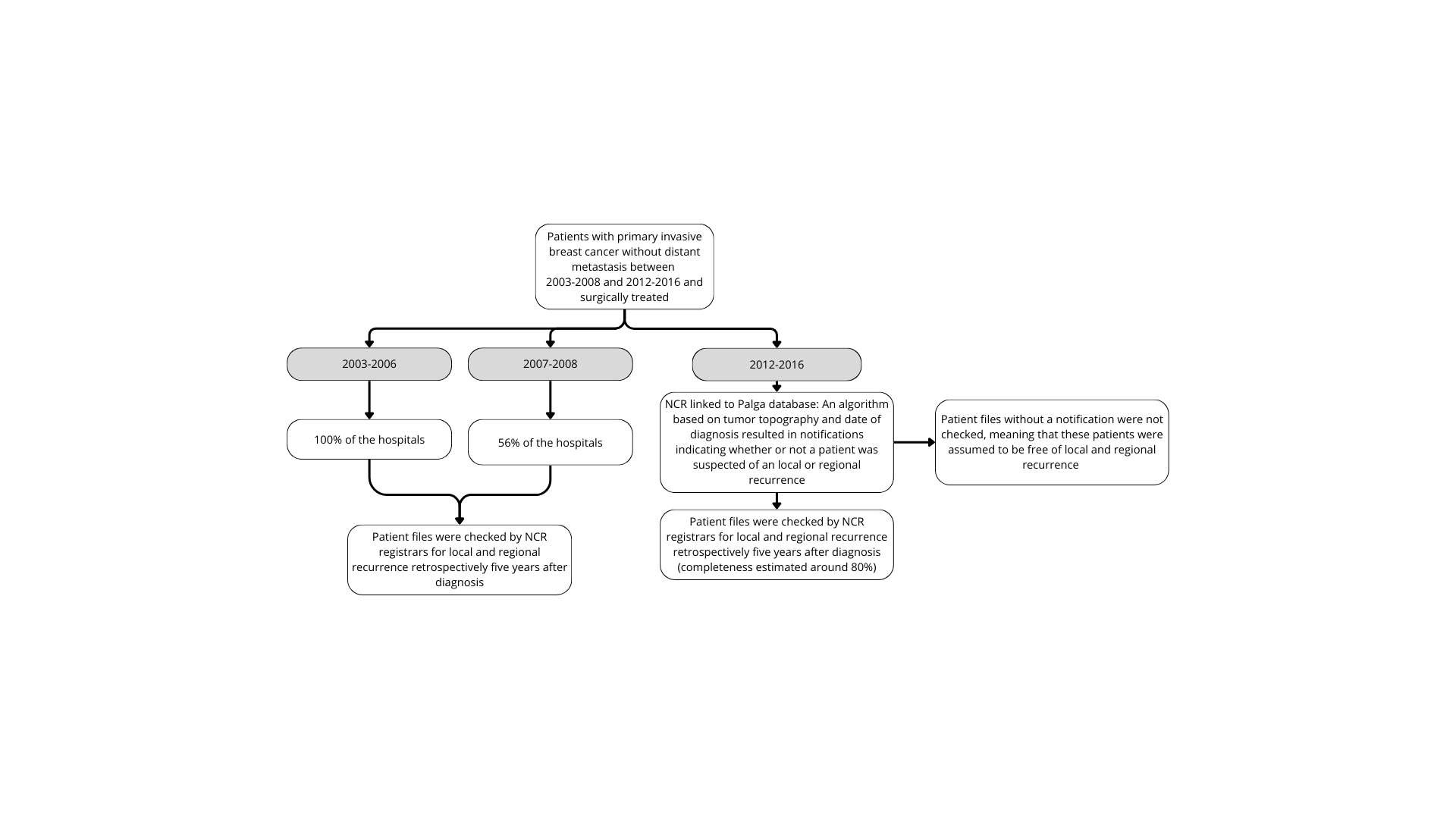 |
| --- |
| **Supplementary Figure A:** Flowchart of data collection of LR and RR in the two specified time periods.  Patients with multiple recurrences ≤90 days of the first recurrence were counted in multiple recurrence groups.  The number of LRs and RRs in 2012-2016 were corrected by dividing the number of LRs and RRs in every year between 2012 and 2016 by four and then multiplying by five. The proportion of ipsilateral second primary tumors also in the LR group is not included in the correction here, as the number of second primary tumors is complete in the NCR. |

| \|  \| Number of recurrences (N=121347) \| \| \| \| \| \| \| \| \| \| \| \| \| \| --- \| --- \| --- \| --- \| --- \| --- \| --- \| --- \| --- \| --- \| --- \| --- \| --- \| --- \| \| 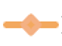 \| 346 \| 314 \| 272 \| 257 \| 153 \| 157 \|  \|  \| 191 \| 153 \| 160 \| 186 \| 160 \| \| 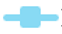 \| 193 \| 189 \| 156 \| 141 \| 88 \| 99 \|  \|  \| 176 \| 158 \| 178 \| 190 \| 170 \| \| 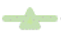 \| 232 \| 204 \| 195 \| 232 \| 124 \| 138 \|  \|  \| 181 \| 193 \| 180 \| 192 \| 195 \| |
| --- | --- | --- | --- | --- | --- | --- | --- | --- | --- | --- | --- | --- | --- | --- | --- | --- | --- | --- | --- | --- | --- | --- | --- | --- | --- | --- | --- | --- | --- | --- | --- | --- | --- | --- | --- | --- | --- | --- | --- | --- | --- | --- | --- | --- | --- | --- | --- | --- | --- | --- | --- | --- | --- | --- | --- | --- |
| **Supplementary Figure B:** Proportions of breast tumor recurrences per incidence year of primary tumor (N=121347)  LR, local recurrence; RR, regional recurrence; CBC, contralateral breast cancer.  Patients with multiple breast tumor recurrences ≤90 days of the first recurrence were counted in multiple recurrence groups.  * For patients diagnosed in 2007 or 2008, data was only available from 56% of the Dutch hospitals.  Differences in average recurrence rates (average recurrence rate for 2003-2008 cohort compared to average recurrence rate for 2012-2016 cohort) between cohorts were tested with two-sided unpaired t-test, with a *P-*value of <0.05 being considered significant. |

| \|  \|  \| Number of events (N=63876) \| \| --- \| --- \| --- \| \| 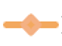 \|  \| 161 134 127 113 64 64 88 68 65 80 72 \| \| 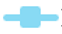 \|  \| 80 92 72 68 44 41 71 73 74 81 78 \| \| 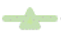 \|  \| 118 102 110 119 60 72 115 107 95 114 110 \|  \|  \|  \| Number of events (N=28292) \| \| --- \| --- \| --- \| \| 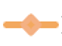 \|  \| 110 103 78 88 51 36 33 35 34 43 36 \| \| 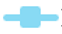 \|  \| 78 65 51 45 29 26 46 44 65 55 51 \| \| 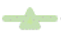 \|  \| 63 55 37 58 31 32 27 29 27 27 20 \|  \|  \|  \| Number of events (N=29179) \| \| --- \| --- \| --- \| \| 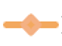 \|  \| 75 77 67 56 38 57 70 50 61 63 52 \| \| 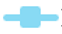 \|  \| 35 32 33 28 15 32 59 41 39 54 41 \| \| 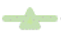 \|  \| 51 47 48 55 33 34 39 57 58 51 65 \| |
| --- | --- | --- | --- | --- | --- | --- | --- | --- | --- | --- | --- | --- | --- | --- | --- | --- | --- | --- | --- | --- | --- | --- | --- | --- | --- | --- | --- | --- | --- | --- | --- | --- | --- | --- | --- | --- |
| **Supplementary Figure C:** Proportions of breast cancer events per incidence year of primary tumor and per age group (N=121347)  LR, local recurrence; RR, regional recurrence; CBC, contralateral breast cancer.  Patients with multiple breast tumor recurrences ≤90 days of the first recurrence were counted in multiple recurrence groups.  * For patients diagnosed in 2007 or 2008, data was only available from 56% of the Dutch hospitals.  Differences in average recurrence rates (average recurrence rate for 2003-2008 cohort compared to average recurrence rate for 2012-2016 cohort) between cohorts were tested with two-sided unpaired t-test, with a *P-*value of <0.05 being considered significant. |

| \|  \|  \| Number of events (N=57565) \| \| --- \| --- \| --- \| \| 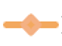 \|  \| 105 98 97 88 57 56 72 52 74 72 64 \| \| 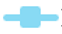 \|  \| 52 50 50 45 27 26 61 46 53 52 57 \| \| 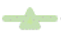 \|  \| 126 104 119 130 75 80 121 135 111 133 140 \|  \|  \|  \| Number of events (N=48323) \| \| --- \| --- \| --- \| \| 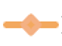 \|  \| 150 124 103 98 59 65 84 63 57 73 65 \| \| 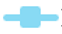 \|  \| 93 86 72 53 38 42 79 67 79 87 75 \| \| 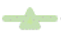 \|  \| 75 76 51 72 34 42 40 38 54 45 43 \|  \|  \|  \| Number of events (N=15459) \| \| --- \| --- \| --- \| \| 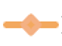 \|  \| 91 92 72 71 37 36 35 38 29 41 31 \| \| 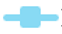- \|  \| 48 53 34 43 23 31 36 45 46 51 38 \| \| 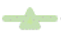 \|  \| 31 24 25 30 15 16 20 20 15 14 12 \| |
| --- | --- | --- | --- | --- | --- | --- | --- | --- | --- | --- | --- | --- | --- | --- | --- | --- | --- | --- | --- | --- | --- | --- | --- | --- | --- | --- | --- | --- | --- | --- | --- | --- | --- | --- | --- | --- |
| **Supplementary Figure D:** Proportions of breast cancer events per incidence year of primary tumor and per primary tumor stage (N=121347)  LR, local recurrence; RR, regional recurrence; CBC, contralateral breast cancer.  Patients with multiple breast tumor recurrences ≤90 days of the first recurrence were counted in multiple recurrence groups.  * For patients diagnosed in 2007 or 2008, data was only available from 56% of the Dutch hospitals.  Differences in average recurrence rates (average recurrence rate for 2003-2008 cohort compared to average recurrence rate for 2012-2016 cohort) between cohorts were tested with two-sided unpaired t-test, with a *P-*value of <0.05 being considered significant. |

| \|  \|  \| Number of events (N=51326) \| \| --- \| --- \| --- \| \| 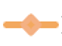 \|  \| 111 124 105 89 60 48 66 51 65 74 72 \| \| 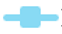 \|  \| 51 52 55 38 31 27 39 40 46 69 67 \| \| 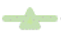 \|  \| 106 100 75 99 59 62 71 60 71 70 77 \|  \|  \|  \| Number of events (N=26459) \| \| --- \| --- \| --- \| \| 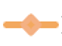 \|  \| 42 23 25 36 24 25 32 27 22 27 28 \| \| 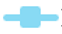 \|  \| 10 13 4 14 4 7 15 11 12 15 17 \| \| 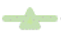 \|  \| 55 39 56 51 34 38 52 74 58 73 78 \|  \|  \|  \| Number of events (N=33243) \| \| --- \| --- \| --- \| \| 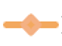 \|  \| 159 141 115 104 53 63 75 53 51 67 54 \| \| 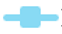 \|  \| 111 109 82 78 46 55 98 80 83 82 77 \| \| 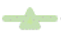 \|  \| 58 50 44 60 23 28 39 40 36 39 33 \| |
| --- | --- | --- | --- | --- | --- | --- | --- | --- | --- | --- | --- | --- | --- | --- | --- | --- | --- | --- | --- | --- | --- | --- | --- | --- | --- | --- | --- | --- | --- | --- | --- | --- | --- | --- | --- | --- |
| **Supplementary Figure E:** Proportions of breast cancer events per incidence year of primary tumor and per primary tumor grade (N=111028)  LR, local recurrence; RR, regional recurrence; CBC, contralateral breast cancer.  Patients with multiple breast tumor recurrences ≤90 days of the first recurrence were counted in multiple recurrence groups.  * For patients diagnosed in 2007 or 2008, data was only available from 56% of the Dutch hospitals.  Differences in average recurrence rates (average recurrence rate for 2003-2008 cohort compared to average recurrence rate for 2012-2016 cohort) between cohorts were tested with two-sided unpaired t-test, with a *P-*value of <0.05 being considered significant. |

| \|  \|  \| Number of events (N=8448) \| \| --- \| --- \| --- \| \| 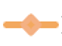 \|  \| 26 20 9 12 16 12 14 16 11 \| \| 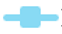 \|  \| 16 7 7 10 10 15 14 14 10 \| \| 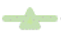 \|  \| 11 11 7 5 15 7 7 6 10 \|  \|  \|  \| Number of events (N=71354) \| \| --- \| --- \| --- \| \| 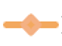 \|  \| 118 131 83 82 112 85 100 105 90 \| \| 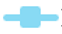 \|  \| 67 70 37 46 81 74 70 96 85 \| \| 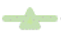 \|  \| 120 150 94 102 120 143 134 140 163 \|      \|  \|  \| Number of events (N=10751) \| \| --- \| --- \| --- \| \| 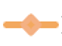 \|  \| 50 62 39 42 48 42 35 46 39 \| \| 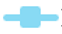 \|  \| 34 41 28 28 66 55 74 63 50 \| \| 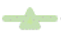 \|  \| 24 36 17 16 27 25 24 30 14 \|  \|  \|  \| Number of events (N=4552) \| \| --- \| --- \| --- \| \| 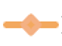 \|  \| 28 21 12 16 9 8 6 9 12 \| \| 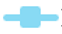 \|  \| 15 12 10 11 14 11 15 14 19 \| \| 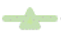 \|  \| 7 16 2 10 7 10 7 6 3 \| |
| --- | --- | --- | --- | --- | --- | --- | --- | --- | --- | --- | --- | --- | --- | --- | --- | --- | --- | --- | --- | --- | --- | --- | --- | --- | --- | --- | --- | --- | --- | --- | --- | --- | --- | --- | --- | --- | --- | --- | --- | --- | --- | --- | --- | --- | --- | --- | --- | --- |
| **Supplementary Figure F**: Proportions of breast cancer events per incidence year of primary tumor and per primary tumor subtype (N=95105)  LR, local recurrence; RR, regional recurrence; CBC, contralateral breast cancer.  Only data from 2005-2008 and 2012-2016 were included in the analyses by breast cancer subtype, as there was no available data in the NCR on breast cancer subtype for 2003-2004.  Patients with multiple breast tumor recurrences ≤90 days of the first recurrence were counted in multiple recurrence groups.  * For patients diagnosed in 2007 or 2008, data was only available from 56% of the Dutch hospitals.  Differences in average recurrence rates (average recurrence rate for 2003-2008 cohort compared to average recurrence rate for 2012-2016 cohort) between cohorts were tested with two-sided unpaired t-test, with a *P-*value of <0.05 being considered significant. |
